# Students’ attitudes towards somatic genome editing versus genome editing of the germline using an example of familial leukemia

**DOI:** 10.1007/s12687-021-00528-1

**Published:** 2021-05-08

**Authors:** Beate Vajen, Joelle Ronez, Wiebke Rathje, Laura Heinisch, Smilla Ebeling, Ulrich Gebhard, Corinna Hößle, Brigitte Schlegelberger

**Affiliations:** 1 Department of Human Genetics, Hannover Medical School, Hannover, Germany; 2 Institute for Biology and Environmental Science, Carl-von-Ossietzky University Oldenburg, Oldenburg, Germany; 3 Faculty of Education, University Hamburg, Hamburg, Germany

**Keywords:** Somatic genome editing, Genome editing of the germline, Medical students, Moral attitudes

## Abstract

Although the discussion on possibilities and pitfalls of genome editing is ever present, limited qualitative data on the attitudes of students, who will come into contact with this technology within a social and professional context, is available. The attitude of 97 medical students and 103 students of other subjects from Hannover and Oldenburg, Germany, was analyzed in winter 2017/18. For this purpose, two dilemmas on somatic and germline genome editing concerning familial leukemia were developed. After reading the dilemmas, the students filled out a paper-and-pencil test with five open questions. The qualitative evaluation of the answers was carried by a deductive-inductive procedure of content analysis. There was a high approval for the use of somatic genome editing. When it came to germline genome editing, concerns were raised regarding enhancement, interventions in nature, and loss of uniqueness. The students recognized that somatic genome editing and germline genome editing prove different ethical challenges and need to be judged separately. Many students expressed not feeling fully informed. The results of this project show the importance of educating the public about the possibilities, limitations, and risks of somatic and germline genome editing. We recommend that this should already be addressed in schools in order to optimally prepare students and adults for participation in public discourse. Especially for patients affected by genetic diseases, it is of great importance that the treating physicians and geneticists are sufficiently informed about the method of genome editing to ensure good counseling.

**Supplementary Information:**

The online version contains supplementary material available at 10.1007/s12687-021-00528-1.

## Introduction

Scientists have had the tools to modify the genomes of organisms since the 1970s. However, the emergence of the CRISPR-Cas technology in 2012 marks a decisive breakthrough in genome editing because of its ability to introduce precise, time-saving, and inexpensive genetic changes into the human genome (CRISPR: clustered regularly interspaced short palindromic repeats, Cas: CRISPR-associated) (Jinek et al. 2012; Doudna and Charpentier 2014). Diseases caused by pathogenic variants of a single gene can be treated causally only by gene correction. This appears to be particularly simple in the case of autosomal recessive diseases in which both gene copies inherited from the father and the mother are defective such as in the case of cystic fibrosis (Mention et al. 2019). Genome editing can be applied not only to somatic cells but also to cells of the human germline, such as sperm or oocytes or early embryos. The most significant difference between these two strategies lies in the consequences: a gene alteration caused by somatic genome editing is usually not passed on to offspring. Genome editing of the germline inevitably leads to heritability of the introduced genes and, thus, also affects the offspring. The goal of genome editing by the CRISPR-Cas technology is to introduce functioning genes into specific cells.

In the first step, the Cas proteins generate a DNA double-strand break. The resulting DNA double-strand is repaired by the error-prone repair mechanism of non-homologous end-joining (Miyaoka et al. 2016), which often results in genetic insertions or deletions. Although specific guide RNAs allow the Cas proteins to bind to their target DNA sequence with high accuracy, genetic changes can also be introduced at other unintended locations in the genome as so-called off-target effects. Homology-directed repair (HDR) is an alternative repair mechanism, that allows error-free repair (Zaboikin et al. 2017) in which the cell uses an intact copy of the DNA sequence as a template. This mechanism is used in genome editing approaches for an error-free correction of genetic changes (Jinek et al. 2012). In the meantime, numerous clinical studies have been initiated and are still ongoing with the aim of developing a causal treatment of genetically determined diseases by means of somatic genome editing (Hu 2017; Lu 2016; Wu 2017).

Modifications of the human genome pose ethical challenges. Their ethical evaluation requires a close dialogue between science and the public. The birth in November 2018 of genetically modified twin sisters by CRISPR-Cas9 technology in China underscores the urgency of understanding how to responsibly address the issue of germline interventions (Cyranoski and Ledford 2018; Li et al. 2019; Wang et al. 2018). The inventors of the CRISPR-Cas technology therefore call for a global moratorium on all clinical uses of human germline editing (Lander et al. 2019). In their *Nature Genetics* editorial on the future of genome editing, a committee consisted of research scientists, clinicians, regulatory agents, and bioethicists has pointed out the importance of informing the public about the risks and opportunities of the new CRISPR-Cas technology (the future of human genome editing 2017). The German Research Association, the German Academy of Engineering Sciences, and the National Academy of Science Leopoldina have published a joint statement in 2015 (German Research Society 2015). In their recently published position paper on the question of human germline genome editing, the German Ethics Council has addressed the issue of human germline editing (German Ethics Council 2019). The German Ethics Council does not consider the human germline to be inviolable, but it currently assesses germline interventions as ethically irresponsible because of their incalculable risks.

Public attitudes towards gene editing for human use have been previously reviewed (Delhove et al. 2020). Delhove et al. (2020) have reported the most common ethical concerns in the public to be “genome editing interferes with nature” and “genome editing is like playing God.” Genome editing for the treatment of diseases has been found to be more morally accepted compared to enhancement (Critchley et al. 2019; Gaskell et al. 2017; Scheufele et al. 2017; Wang et al. 2017). Furthermore, acceptance of germline genome editing is generally lower than for somatic genome editing (Iredale et al. 2003). Most studies were conducted in the USA and follow a quantitative study design (Howell et al. 2020). Only limited qualitative data of the attitudes of medical students, who will come into contact with this method in a professional and social context, is available. We developed two medical dilemmas based on the current medical practice with a personal point of view to address the students directly. The aim of this interdisciplinary project was to investigate the following questions: How do students rate the use of somatic genome editing? Are there differences in the moral position of students when using germline genome editing? What arguments do they use to express their opinion and does the subject or gender of the respondents influence their moral position?

## Method

### Research team and reflexivity

B.V. (female) conducted a qualitative study in winter 2017/18 in Hannover, Germany, and Oldenburg, Germany, to explore the moral positions of 200 students. The students were informed that B.V. is a Biologist and worked as a research associate at the Department of Human Genetics, Hannover Medical School, at the time of the study. Before the students were presented with the dilemmas and questionnaires, B.V. informed them about a new technology called genome editing that could be used to change the genetic material and eliminate inherited genetic diseases in the future. She informed the students about the interdisciplinary project between the Department of Human Genetics in Hannover, the Institute for Biology and Environmental Science, Oldenburg, and the Faculty of Education, Hamburg, funded by the German Federal Ministry of Education and Research, and that one aim of this project was to find out the students' attitude towards genome editing.

### Study design

The participants were asked face-to-face to participate. The participation was voluntary and anonymous. The number of medical students (97) and students from other subjects (103) was comparable, while the number of female students was significantly higher than the number of male students. The numbers of students of the different subjects are listed in the Appendix (Table S1). The students, who agreed to participate in the study, were asked to answer all questions. Partly unanswered questionnaires were not included in the analysis (8%). After excluding these questionnaires, the response rate was 92%.

A qualitative research approach was chosen for this study because they are particularly suited to answering open-ended research questions. The aim of this study was to describe the subject matter. Therefore, no hypotheses were formed before collecting the data. Students were asked to express their opinions freely as suggested by Porst (2014). According to Döring (Döring and Bortz 2016), the qualitative questionnaire is the counterpart to the interview guide in semi-structured oral interviews. We refrained from interviews or group discussions because these methods do not allow precise phrasing or sorting of the questions. Therefore, a non-standardized questionnaire was developed that allowed the collection of qualitative data. This way, comparable data were collected. Furthermore, this validated questionnaire can be made available to other researchers in the future and is suitable for systematic repetition. Furthermore, greater anonymity was provided here compared to the interview and respondents had more time to consider their answers.

One disadvantage of the questionnaire method is that there is no opportunity for follow-up questions. The researcher was not able to ask any further questions due to the anonymization for example if the writing was not legible. Another limitation is that the dilemma stories and the following questions require a good understanding of the text. Furthermore, a high motivation was necessary to answer the free text tasks in detail. As a result, some of the questions remained unanswered. These questionnaires were excluded. No repeat interviews were carried out. The data were not recorded. The data were collected at the workplace. No one else was present apart from the participants and the researcher. The questionnaire consisted of three sections (the original questionnaires are attached in the Appendix). A written dilemma (either on somatic or germline genome editing) was presented in the first section, (dilemmas are attached in the Appendix). The participants were asked to read them. The second section contained a questionnaire with open questions inspired by Visser and Hößle (2015) for the moral evaluation of the situation described in the dilemma. In the third section, the participants were asked to answer questions regarding their person. Age, gender, and the subject of study were collected. Furthermore, the participants were asked to generate a code for anonymization of the questionnaire.

Two different fictitious dilemmas were developed to analyze the moral position of the students with regard to somatic genome editing (dilemma 1) versus germline genome editing (dilemma 2). These dilemmas were based on the definition of Rehfus (2003), in which a situation offers two possibilities, neither leading to a preferable or desirable solution. Each dilemma had a personal point of view and considered the target group as it has been suggested by Bögeholz et al (2007).

Dilemma (1) describes a situation in which a young couple is talking about an uncle with a pathogenic variant in the *RUNX1* gene. Because of this pathogenic variant, the uncle has an increased chance of developing leukemia. His clinically healthy son, who also carries the pathogenic variant, could be one of the first children in Germany in whom this genetic change would be corrected using somatic genome editing. The dilemma also contains a brief explanation of the method: “The gene that is responsible for leukemia development is simply cut out from the DNA of removed bone marrow stem cells and replaced with an intact gene. The defective cells in the body are then destroyed by a pre-treatment and the edited cells are returned to the body. This means that the risk of developing leukemia is no longer increased. The editing does not apply to Julian's future children, as Julian's germ cells are not affected.”

Dilemma (2) describes a situation in which a young couple is talking about an uncle with a pathogenic variant in the *RUNX1* gene. Here, the uncle would like to become a father and does not rule out germline genome editing. His wife could give birth to the first German child whose genome was altered using germline genome editing by correcting the pathogenic variant of the *RUNX1* gene. The dilemma also contains a brief explanation of the method: “First, an egg cell from Ulrike is artificially fertilized with the sperm cells from Wilhelm. In a second step, the gene responsible for the leukemia is cut out of the embryo’s DNA and is replaced with a new gene. This means that the risk of developing leukemia is low. The best part is that it also applies to future generations. Uncle Wilhelm no longer needs to worry that he will pass on his leukemia predisposition to his grandchildren”.

Based on personal feedback in a pre-test with 20 students, the question design was optimized and interfering factors such as social desirability and mean value orientation were minimized. Additional professional information about leukemia was omitted, because the students perceived the dilemmas as to long and the additional information was considered confusing. To reduce the possibility of contrasting effects, every participant received only one dilemma.

After reading the dilemma (either (1) or (2)), the students were asked to answer five questions:

1. Is there an ethical problem, if so what kind of ethical problem?
2. Which values play a role in this situation?
3. What could be the consequences, if genome editing was carried out?
4. Who or what does genome editing affect?
5. Would you decide for or against genome editing?

The questions were structured to test the sub-competencies “perceiving and becoming aware of moral relevance,” “judging,” “consequence reflection,” “change of perspective,” and “judging” (Visser and Hößle 2015). Verbal consent was obtained. Students were asked to justify their answers in detail. It took the students between 20 and 45 min to complete the questionnaires.

Sixty-one medical students received the questionnaires with the dilemma (1), in which somatic genome editing to prevent familial leukemia was described. Dilemma (2) with the topic of germline genome editing was given to 36 medical students and to 39 students of other subjects.

### Participants

Sample selection was based on theoretical sampling. Thus, the subjects were not selected randomly, but based on preliminary considerations. All students had obtained the general German university entrance qualification since basic genetic knowledge is included in the Lower Saxony Core Curriculum of grade 10. The topic of genome editing is not included in the Curriculum.

### Data evaluation

The questionnaires were digitized and transcribed. Answers were not edited since they were available in text form. Answers were grammatically changed only when they were used as direct quotations in the manuscript. There was no paraphrasing and segregation to prevent falsification of the statements (Döring and Bortz 2016). MAXQDA 2018 software was used to support data analysis. The qualitative evaluation of the answers was carried out according to the content analysis of Mayring (2015). The following steps were carried out: definition of the analysis material, definition of the structuring dimensions, determination of main categories and subcategories (research-led, deductive), formulation of definitions and examples, review of questionnaires and coding of the data, revision and differentiation of the categories (material-led, inductive), preparation of the results. In the work at hand, a deductive-inductive procedure as suggested by Döring and Bortz (2016) was chosen. Consequently, on the basis of the state of research, a rough formation of categories was already carried out before the survey was conducted. After reviewing the collected questionnaire data, these categories were differentiated and supplemented as suggested by Kuckartz (2018). The entire questionnaire was analyzed. The following quality criteria according to Mayring (2015) were met: (1) Intercoder reliability—to ensure objectivity, a second coder (J.R.) was consulted. The researchers agreed on a common approach for coding the data. An intersubjectively comprehensible analysis of the results was ensured due to the created category system. The categories were discussed with all coauthors. (2) Intracoder reliability—the text was coded twice with a time interval, to ensure that the same categories were assigned to the text passages. (3) Procedural documentation—the documentation was carried out in the way that the analysis is understandable and comprehensible to others. (4) Rule guidance—the evaluation was defined accurately and carried out systematically to ensure the quality of result interpretation. (5) Proximity to the object—with the presentation of dilemmas in which a young couple is talking about genome editing, we established a reference to the everyday world of the students. A communicative validation as suggested by Mayring (2015) could not be carried out due to the anonymous survey. For B.V. and J.R., saturation was reached; no further data could be collected or new categories created.

## Results

### Participant characteristics

A total of 97 medical students and 103 students of other subjects participated in this study. Table 1 shows the sample characteristics of all participants. The group of medical students included 32 (33%) male and 65 (67%) female students with a mean age of 20. Twenty-seven of the participants in the group of students of other subjects were (26%) male students and 76 (74%) were female students with a mean age of 22. The respective numbers of students of the different subjects are listed in the Appendix (Table S1).

**Table 1:** Number and characteristics of the participants

Group	Medical students		Students of other subjects*	
Gender	Male	Female	Male	Female
	32 (33%)	65 (67%)	27 (26%)	76 (74%)
Mean age (with standard deviation)	20 ± 3.89		22 ± 3.32	
Total number	97		103	

*Other subjects included the following: biochemistry, biomedicine, biotechnology, history, teaching, management, philosophy, political science, psychology, theology, and veterinary medicine

### Students’ attitudes toward somatic genome editing

In general, the students independent of the subject of the study had favorable attitudes towards somatic genome editing. Students put great hope in this novel method to prevent severe diseases. Many respondents did not see any moral problem with somatic genome editing. However, their answers revealed only little reflections of possible long-term medical or social consequences following somatic genome editing. This became clear from the answers to the question “Who or what does genome editing affect?” because many students thought that somatic genome editing affects only the treated person and no one else. Students, who gave their answers in more detail, primarily focused on the topic of “health.” They often felt the moral obligation to do everything in their power to help all patients. Yet, often a counter-argument was made as to the potential higher risk that somatic genome editing might pose to the health of all patients in comparison to conventional therapies. They raised concerns that changing the genome may have unforeseen consequences. Again, they felt the moral obligation to protect patients by giving preference to therapies with known side effects over genome editing with unknown long-term consequences:

Is reducing the risk of a potentially life-shortening disease so important that it is acceptable to use a procedure that has not been fully researched with respect to long-term effects? (PA15, student of Education, female)

Arguments related to health could not always be attributed to supporters or opponents of genome editing. Interestingly, the value “health” was a common reference point for both viewpoints, of those who supported or disapproved of somatic genome editing. While the proponents of genome editing emphasized the therapeutic possibilities of previously incurable diseases, the opponents focused on possible health risks of the method:

I see genome editing as a great opportunity to save lives and reduce suffering from disease" (BP08, student of Biomedicine, male) versus "I think it’s risky to treat people with it. Although it is a good idea to eliminate diseases with it, one can also possibly influence much more than actually intended. Possibly new mutations or defects can arise and have worse effects than before. (HH01, student of Biomedicine, female)

In addition to the desire for clear rules for the use of somatic genome editing and research into long-term consequences, the costs of the therapy were often pointed out. They demanded that the costs should not be an obstacle to access to the therapy. The students often expressed their need for further information on genome editing technology:

Since I currently have far too little knowledge of genome editing, especially regarding the risks, I would decide against it. Basic education would be very desirable." (NJ08, student of Biomedicine, male) “Formulating an exact opinion is not possible for me with the current state of knowledge. I would like to suggest that schools also discuss CRISPR and so on in more detail to enable someone like me to make a differentiated judgment. (KG11, medical student, male)

### The use of germline genome editing raised concerns about creation of designer babies, intervention in nature, and an increase in social conflicts

This second dilemma evoked far more intense conflicts compared to the answers regarding somatic genome editing. Nonetheless, around half of the students of all subjects were in favor of germline genome editing. Students considered germline genome editing an ethical problem. The students were asked to name the values that play a role in the dilemma concerning germline genome editing. Here, the following values were named very often: health, dignity of a human being, respect, acceptance, perfectionism, responsibility, and empathy. Without exception, at least one social consequence was named. When analyzing the detailed answers given, it became clear that the overall concerns were greater compared to somatic genome editing. The fear that germline genome editing may pave the way to enhancement, that it represents an interference in nature and God’s creation, and that it leads to the loss of uniqueness and the emergence of a two-class society of the healthy and the sick was mentioned most frequently. The following excerpts demonstrate these fears:

#### Students are concerned about the creation of designer babies

Students fear the use of the method for the purpose of enhancement of unborn children as soon as the method becomes available:

I would definitely decide against it, because this form of genome editing could lead to the creation of so-called "designer babies". From a moral point of view, this is not justifiable, since every human being should be accepted and loved, no matter what they look like. Even if primarily only the disease is to be fought, I think it would still come down to that later. (KR07, medical student, female)*.*

The fear of enhancement is, therefore, unreservedly linked to the call for control and legal guidelines. Furthermore, parents of affected children should be counseled and educated.

For me to be in favor of genome editing, there would have to be very clear requirements that regulate this process—similar to abortion. It should be legally defined in which cases genome editing can/should be done. Comprehensive education of the parents would also have to take place. (BF06, student of Biomedicine, male).

The students often associated science fiction scenarios with this dilemma:

As soon as a manipulation/alteration of the genetic material would be legalized, it could be exploited to “create” better, stronger, more intelligent etc. humans. Life would come only from Petri dishes. It resembles the building and the installation of a robot. (BG12, student of Vocational Education, female).

It is also noteworthy that some students considered society as very ruthless and use the slippery-slope argument:

I would decide against genome editing in any case, because it would cross a line that would be unmanageable! If this step is taken, the respect of human beings and the individuality of every human being is ommitted! It would forcefully evolve into a zero-tolerance society with categorical groups. Parents would be blamed for untreated diseases. Religious values would fade into the background. Respect for the individuality of every human being and, thus, also of diseases must be preserved in favor of a tolerant society… So definitely: NO! (CM10, medical student, female).

#### Students are concerned about interventions in nature leading to loss of uniqueness

Some students perceived genome editing as a presumptuous intervention in nature or God’s creation:

On the other hand, the question arises to what extent man may intervene in the fate of another human being and seize control over the genome. In principle, this means that he is interfering with nature. (IA01, student for Education, female). I would decide against genome editing, because we are a creation of God and one should not interfere with this. We are images of God and if we change ourselves genetically (for the better), we question God's wisdom/power. (KA03, medical student, female).

They feared the loss of individual traits by intervening with nature and praised the diversity of our current society. Genetic uniformity was not perceived as a desirable goal:

Furthermore, such an intervention would reduce the diversity of our society, which is precisely what makes our lives so colorful. Because through genome editing we would follow an ideal. The individuality of the human being has no more value. (US07, student for Education, female).

In particular, the medical students often suggested that illness or disability should be viewed as regarded traits that create a society with unique individuals. The unconditional acceptance of sick children, too, was mentioned in the context of naturalness and uniqueness:

I would decide against genome editing. Especially when a couple decides to start a family, I think that unconditional love for the unborn child should be in the foreground. Sure, as a parent you worry that something might happen to the unborn, but I personally want to accept my child unconditionally and not 'create' it according to my ideas. (VM09, medical student, male).

#### Students are concerned about an increase in social conflicts

When describing the effects of germline genome editing on society, it was often mentioned that a two-class society could develop. Either due to financial reasons, since not all those affected can afford genome editing, or due to the exclusion of sick children that could have been cured by genome editing. An increasing intolerance in dealing with the sick and physically handicapped was feared:

On the other hand, a major moral problem arises from the statement that is made with the application and also development of this technology: There is no place for illness in this world. Thus, sick people are considered unacceptable, which is highly problematic. (AM02, student of Special Education, female).

Students also pointed out that germline genome editing could lead to conflicts within the family:

Such a “predisposed” life comes with many burdens and reproaches. From the beginning, one is compelled to be grateful and happy. This can cause discord and quarrels within the family. This is the opposite of the intended goal. (PK06, medical student, male).

Furthermore, alternatives to genome editing such as adoption or the admission of foster children were mentioned in this context and the medical need for germline genome editing was questioned here:

Our society is always on the way to becoming perfect and changing the genome could further strengthen the desire for perfect children. However, in my opinion, a human being is characterized by the fact that he or she is not perfect. Of course, it is bad for the families that are concerned if there is a massive hereditary disease, but there would be other possibilities for a family, e.g., adoption or foster children. (EH11, medical student, female).

## Discussion

This study demonstrates that students independent of their subject of study generally supported the usage of genome editing in general. The students recognized the potential of the technology and mentioned benefits to individuals or society. However, students were not unreservedly in favor of using this technology. They called for clear rules as to the conditions under which this technology may be used. Furthermore, they would only approve its application if potential complications or long-term consequences could be better estimated. Since the moral conflicts that result from the application of somatic and germline genome editing were perceived differently, we discuss both topics separately.

### Students’ attitudes towards somatic genome editing

Somatic genome editing received strong support in this study. This is in line with previous studies that have analyzed the public opinion (also including medical students) towards somatic genome editing (Iredale et al. 2003). Recent studies have reported that genome editing was perceived more positive amongst males and younger people and also in people that have shown more trust in science (Critchley et al. 2019; McCaughey et al. 2016). In line with the mentioned studies, we did observe slight differences between the decisions made by male or female students. Male students were in favor with genome editing more often. Furthermore, the demographic disparities are likely to be influenced by socio-political and cultural factors that may differ between the countries in which the studies have been conducted. Often students did not recognize any ethical conflict. To them, dilemma (1) dealing with somatic genome editing did not evoke any ethical conflict. Students’ also only rarely explained their decision for or against somatic genome editing in more detail compared to the answers given to the dilemma (2) dealing with germline genome editing. Interestingly, effects on society as a whole were only rarely mentioned. This suggests that, regardless of the subject of study, students regarded somatic genome editing as a feasible preventive option without ethical problems. The ethical evaluation of somatic genome editing was made in terms of an opportunity and risk assessment. In its ethical assessment of somatic gene therapy, the German Reference Center for Ethics in the Biosciences (DRZE) also has focused on the general conditions of clinical research than on the possible social and ethical consequences. The DRZE only mentioned ethical challenges addressing starting points of studies, reasons for study interruptions or restarts, and the selection of participants (Baum et al. 2013).

For a large number of diseases that could be treated by genome editing, there is no need to edit the germline; it is sufficient to change somatic cells only (Lundberg and Novak 2015). Since the students mentioned that they would like to have more information on the legal framework for somatic genome editing, it seems reasonable to educate and provide information about regulations that already exist in Europe and the USA. This seems all the more important since genome editing will rapidly find its way into the treatment of genetically determined diseases in the near future. To date, the US American FDA has approved twenty genome editing products (FDA 2021). In addition, 671 clinical studies using gene therapy are currently listed (ClinicalTrials 2021).

The most mentioned fear of the usage of the CRISPR-Cas9 technology was the fear of possible side effects that genome editing might have. Recent publications have shown that the widespread CRISPR-Cas9 technology leads to off-target effects with far-reaching consequences. It has been shown in various cancer cell lines that the expression of Cas9 can lead to the emergence and expansion of inactivating mutations of tumor suppressor genes, e.g., *TP53* (Enache et al. 2020). To rule out such off-target effects, methods have been developed that greatly reduce changes in the genome at unintended locations (Grunewald et al. 2019), including prime editing, where off-target effects are reduced by introducing a DNA single-strand break and an increased efficiency of integrating the template DNA (Anzalone et al. 2019). Overall, the students showed high agreement with the technology of somatic genome editing regardless of the subject of study. However, the students call for security of the method, legal framework conditions, control of costs, and access to this therapy for all patients.

### Students’ attitudes towards germline genome editing

More students decided against genome editing when the dilemma focused on germline genome editing. This was the case for students of all subjects. Interestingly, Armsby et al. (2019) have shown a similar effect of members of genetics professional societies. Almost all members that have been inquired were in favor of using somatic genome editing, but only half of them were supportive of germline genome editing. Likewise, the students that participated in our study were often concerned about enhancement. This is in line with studies focusing on public attitudes, which have reported an overall high acceptance to increase human health but have shown only low public support to enhance attributes or appearance in healthy humans (Critchley et al. 2019; McCaughey et al. 2016). In a survey across 11 countries, Gaskell and colleagues (Gaskell et al. 2017) have asked members of the general public for their willingness to support treatment and enhancement in adults and unborn children. There has been consistently greater support for treatment than for enhancement and for the intervention in adults than in unborn children. This strong opposition towards enhancement was also observed in a Dutch adult population in 2018 (van Dijke et al. 2021). The study by van Dijke et al. (2021) has concentrated on germline genome editing and has shown that the possibility to treat congenital abnormalities positively influenced the attitude towards genome editing. Intervention in the human germline for non-preventive and non-therapeutic purposes (i.e., to only “improve” humans) has clearly been rejected by the European States in the Oviedo Convention (Council of Europe 1997). Some of the students of our study recalled the slippery-slope argument (McGleenan 1995). These students expected that germline genome editing will sooner or later no longer be limited to therapeutic purposes, but that it will be misused to optimize individuals. This concern was not raised when discussing the use of somatic genome editing suggesting that the participating students did not expect similar possibilities for misconduct in this scenario.

Students that were concerned about possible enhancement often focused on the diversity of our society, which for them is worth protecting. They described that physical and mental challenges are part of our society. They feared the loss of uniqueness and authenticity. Students think that people can no longer be themselves if they have been “modified” through genome editing. They stated that there could be a risk of losing personal identity. Students seem to strongly associate their identity with their genes and, thus regard genomic interventions as an intervention in their personality. The argument of loss of authenticity is often mentioned by critics of enhancement technologies (Gaskell et al. 2017). Parens (Parens 2005) has suggested that the critics’ central worry is that these technologies will threaten our efforts at achieving authenticity and will separate us from who we really are.

The idea of fear of unprecedented control of parents over their children’s life, as mentioned by Habermas (2003), was also discussed by some of the students in our study. Habermas points out that modification of the genome will bring a strong asymmetry into the intergenerational relationship and that in consequence genetically manipulated children cannot consider themselves the sole authors of their own lives. The students expected family conflicts that could arise from genome editing initiated by the parents. They assumed that affected children have to be grateful and happy about the decision that their parents made before birth. They mentioned that this “predisposed” life could be a burden. Overall, although the students of this study showed also a high level of agreement with germline genome editing, they have ethical conflicts and name social consequences and fears.

### Study strengths and limitation

Although this study provides valuable insights into the attitudes of students of different subjects on genome editing, the limitations of the study need to be considered. Our anonymous study design did not allow follow-up questions from the participant or the researcher. Repeat interviews or transcripts were not returned to the participants for comments. For deeper analysis (e.g., for analysis of complex relationships), further research with a different setting (e.g., qualitative interviews) is necessary. The strength of this study is the use of medical dilemmas based on the current medical practice with a personal point of view that motivated the students to give personal statements on genome editing. Furthermore, due to different dilemmas on somatic genome editing and germline genome editing, we were able to analyze the different attitudes of students in detail.

## Conclusion

In both scenarios, the students saw a considerable need for further information on both somatic and germline genome editing. This was independent of the subject of study. Thus, also students who will come into contact with this technology in a professional context such as medical students did not feel informed enough. Some of the answers depicted rather unrealistic, fictitious future scenarios. This study showed that the knowledge of genome editing, and perhaps the basic understanding of genetics (monogenic vs polygenic inheritance, influence of epigenetics), needs to be improved in students of all disciplines and in the public in order to make an informed decision about the use of genome editing. In future research, it would also be interesting to re-use these dilemmas to re-examine the attitudes of students after some time, because it has been shown that the positive public opinion towards genome editing began to decrease since 2017 due to negative sentiments derived from news, for instance the reports about the Chinese twins (Müller et al. 2020). We recommend that both the topic of genome editing and the discussion of ethical issues should be intensified in schools. Furthermore, these topics should also be intensified during the study of medicine, biomedicine, or within the training of biology teachers. Especially for patients affected by genetic diseases, it is of great importance that the treating physicians and geneticists are sufficiently informed about the method of genome editing to ensure good counseling.

## Supplementary Information

Below is the link to the electronic supplementary material.

Supplementary file1 (DOCX 31 KB)

## Data Availability

All data can be made available on request.

## Code availability

N.a.
