# Supplementary material for: Students’ attitudes towards somatic genome editing versus genome editing of the germline using an example of familial leukemia
Source: J Community Genet. 2021 May 8;12(3):397–406. doi: 10.1007/s12687-021-00528-1 (PMC8241980; doi:10.1007/s12687-021-00528-1)
Supplement: Supplementary file 1 — Supplementary file1 (DOCX 31 KB) [file 12687_2021_528_MOESM1_ESM.docx]

**Attachment:**

**Questionnaire with dilemma on somatic genome editing**

Paul (18 years old) and Marie (20 years old) are sitting together at the breakfast table and have just learned that Julian (3 years old), the son of Paul's aunt Ulrike and her husband Wilhelm, could be one of the first children in Germany to be genetically modified.

"They were totally shocked to learn that Wilhelm had passed on his leukemia predisposition to their child. This significantly increases the likelihood of developing leukemia. It's nice that there might be a cure this way after all," says Paul.

Marie frowns: "How does genetic modification work anyway? I didn't even know it was possible."

"The whole thing is called genome editing," Paul replies. "The gene responsible for the leukemia is simply excised from the DNA of harvested bone marrow stem cells and replaced with an intact gene. Then the defective cells present in the body are destroyed by pretreatment, and the edited cells are returned to the body. As a result, there is no longer an increased chance of developing leukemia. The editing does not apply to Julian's future children, as Julian's germ cells are not affected. I'm really happy that something like this is now possible."

Marie is not convinced: "So you change a person's DNA in a petri dish? You just can't do something like that!"

"Why not?" replies Paul. "You're doing something good by preventing the outbreak of a serious disease!"

Marie replies, "Where is that supposed to lead? You can't just intervene and change a person. What would happen if genome editing were to change even more than was actually planned? You don't know whether you're really doing something good for humanity in the long run. No, I'm absolutely against something like that!"

1. Is there an ethical problem, if so what kind of ethical problem?

|  |
| --- |
|  |
|  |
|  |
|  |
|  |
|  |
|  |
|  |
|  |
|  |
|  |
|  |

2. Which values ​​play a role in this situation?

|  |
| --- |
|  |
|  |
|  |
|  |
|  |
|  |
|  |
|  |
|  |
|  |
|  |
| 3. What could be the consequences, if genome editing was carried out? |
|  |
|  |
|  |
|  |
|  |
|  |
|  |
|  |
|  |
|  |
|  |

4. Who or what does genome editing affect?

|  |
| --- |
|  |
|  |
|  |
|  |
|  |
|  |
|  |
|  |
|  |
|  |
|  |
|  |
|  |
|  |
|  |
| 5. Would you decide for or against genome editing? |
|  |
|  |
|  |
|  |
|  |
|  |
|  |
|  |
|  |
|  |
|  |

| Gender: | female | male | | |
| --- | --- | --- | --- | --- |
| Subject of study: |  |  |  |  |
| age: | _______ years |  |  |  |

**Questionnaire with dilemma on germline genome editing**

Paul (18 years) and Marie (20 years) are sitting together at the breakfast table and have just found out that Paul's aunt Ulrike and her husband Wilhelm want to give birth to one of the first children in Germany that has been genetically modified.

“They didn't know for a long time whether they should even try to have a child. Because they were so worried, that Wilhelm would pass on his predisposition to leukemia to their child. It's nice that it could work this way,” says Paul.

Marie frowns: “How does something like that work? I didn't even know that it was even possible. "

“It's called genome editing,” replies Paul. “First, Ulrike's egg cell is artificially fertilized with Wilhelm's sperm cells. In a second step, the gene responsible for the leukemia is cut out of the embryo's DNA and replaced with a new gene. This means that the likelihood of developing leukemia is low. The best part is that it also applies to future generations. Ulrike and Wilhelm no longer need to worry that they will pass on their leukemia predisposition to their grandchildren. I'm really happy that something like this is now possible. "

Marie is not convinced: “So you change a person's DNA in a Petri dish? You just can't do something like that! "

"Why not?" answers Paul. "You are doing something good by preventing a serious illness from breaking out!"

Marie replies: “Where is this supposed to lead? You can't just intervene and change someone. What if you change more with genome editing than you actually planned? You don't even know whether you are really doing something good for humanity in the long term. No, I'm absolutely against something like that! "

1. Is there an ethical problem, if so what kind of ethical problem?

|  |
| --- |
|  |
|  |
|  |
|  |
|  |
|  |
|  |
|  |
|  |
|  |
|  |
|  |

2. Which values ​​play a role in this situation?

|  |
| --- |
|  |
|  |
|  |
|  |
|  |
|  |
|  |
|  |
|  |
|  |
|  |
| 3. What could be the consequences, if genome editing was carried out? |
|  |
|  |
|  |
|  |
|  |
|  |
|  |
|  |
|  |
|  |
|  |

4. Who or what does genome editing affect?

|  |
| --- |
|  |
|  |
|  |
|  |
|  |
|  |
|  |
|  |
|  |
|  |
|  |
|  |
|  |
|  |
|  |
| 5. Would you decide for or against genome editing? |
|  |
|  |
|  |
|  |
|  |
|  |
|  |
|  |
|  |
|  |
|  |

| Gender: | female | male | | |
| --- | --- | --- | --- | --- |
| Subject of study: |  |  |  |  |
| age: | _______ years |  |  |  |

**Table S1: Listing of the study programs from the group of students of other subjects**

| **Subject of Study** | **Number of Students** |
| --- | --- |
| Biochemistry | 1 |
| Biotechnology | 1 |
| Teacher training for high schools | 38 |
| Biology | 1 |
| Special education | 3 |
| Biomedicine | 37 |
| Art history | 1 |
| History | 3 |
| Political science | 2 |
| Management | 1 |
| Theology | 3 |
| Philosophy | 1 |
| Veterinary medicine | 1 |
| Psychology | 1 |
| Teacher training for primary schools | 6 |
| Teacher training for vocational schools | 3 |
